# Ipsilateral Nasoseptal Flap Pedicle for Transpterygoid Approach: Technical Note

**DOI:** 10.22038/IJORL.2021.54687.2866

**Published:** 2021-11

**Authors:** Pasquale Anania, Marco Ceraudo, Alessandro Prior, Pietro Fiaschi, Gianluigi Zona, Frank Rikki-Canevari, Diego Criminelli Rossi

**Affiliations:** 1 *Department of Neurosurgery, * *Policlinico San Martino Hospital, IRCCS for Oncology and Neuroscience* *, Genoa, Italy.*; 2 *Department of Neurosciences, Rehabilitation, Ophtalmology, Genetics, Maternal and Children (DINOGMI), University of Genoa, Genoa, Italy.*; 3 *Department of Otorhinolaryngology-Head and Neck Surgery, University of Genoa, Genoa, Italy.*

**Keywords:** Endoscopy, Endonasal, Ipsilateral, Nasoseptal, Reconstruction, Skull base, Transpterygoid

## Abstract

**Introduction:**

Transpterygoid approach is an expanded endonasal approach (EEA) that allows surgical access to the medial infratemporal fossa, to the skull base area of petrous bone and to the Meckel’s cave. During this approach, a sacrifice of sphenopalatine artery is often required, leading to the need of contralateral Hadad-Bassagasteguy flap (HBF) or alternative reconstructive techniques.

**Materials and Methods:**

We report a case of spontaneous CSF leak due to a meningo-encephalocele in the left lateral recess of sphenoid sinus, in which an ispilateral nasoseptal flap was harvest and sphenopalatine artery was preserved.

**Results:**

We described the surgical technique adopted to preserve the ipsilateral nasoseptal vascular pedicle during transpterygoid approach and we performed a review of the pertinent literature.

**Conclusion:**

Wide exposure of the pterygoid base through transpterygoid approach could be obtained preserving the sphenopalatine artery, allowing skull base reconstruction with ipsilateral nasoseptal flap.

## Introduction

Transpterygoid approach (TA) is an expanded endonasal approach (EEA) and a lateral extension of the endonasal corridor that, through the removal of pterygoid process base (1,2), allows surgical access to medial infratemporal fossa, petrous bone, Meckel’s cave and cavernous sinus. During TA, vascular and neural structures, such as vidian nerve and sphenopalatine artery, are encountered and often sacrificed, in order to achieve adequate exposure. The vidian nerve is constituted by parasympathetic fibers going to the sphenopalatine ganglion and the maxillary nerve, which controls lacrimation (3,4). 

It origins from the union of the greater superficial petrous nerve (GSPN) and the deep petrous nerve (from the sympathetic fibers of internal carotid plexus) in the pterygoid canal, entering the pterygopalatine fossa and joining the sphenopalatine ganglion (5). 

The vidian nerve runs in the pterygoid canal(or vidian canal) together with the homonymous artery just laterally to the petrous-paraclival junction of the internal carotid artery (ICA), running in the lateral area of the sphenoid sinus floor, and entering in the pterygopalatine fossa through the vidian foramen, which is situated in the antero-medial portion of pterygoid process base (3,5). The sphenopalatine foramen is located on the base of the pteygoid process, connecting the pterygopalatine fossa and the nasal cavity, and securing the passage of the sphenopalatine artery (terminal branch of the internal maxillary artery), from whose origins the posterior septal artery and its branch: the nasoseptal artery. Preservation of these vascular structures is critical for harvesting a Hadad-Bassagasteguy flap (HBF). The latter, is a vascular pedunculated flap of nasal septum mucosa vascularized by nasoseptal artery, which plays a pivotal role in the reconstructive phase in EEAs (6,7).The vidian canal is used as landmark for petrous and paraclival ICA during TA, and it is often sacrificed during dissection (8,9); vidian nerve lesion could led to eye dryness, with potential risk for corneal ulcers (10). During TA, sphenopalatine artery is often coagulated and cut, leading to the need for contralateral HBF or alternative reconstructive techniques (2,10–15). The aim of this article is to describe the surgical technique for an endoscopic transpterygoid approach while preserving the ipsilateral sphenopalatine artery, in order to use an ipsilateral HBF as reconstructive technique for skull base defects.

## Materials and Methods

We report a case of a patient with acute onset of meningitis, seizures and spontaneous cerebrospinal fluid (CSF) leak due to a left lateral sphenoidal recess meningo-encephalocele. Meningo-encephalocele and CSF fistula repair were performed through a left TA approach and ipsilateral HBF for skull base reconstruction while preserving ipsilateral sphenopalatine artery. We also performed a review of the literature for TA approach using an ipsilateral HBF as closure technique, and we found only five articles describing the preservation of the septal flap pedicle during an ipsilateral TA (8,9,16–18). We describe the surgical technique step by step (Figuer 1) showing the main phases of sphenopalatine artery preservation and vidian nerve identification during TA.

## Results

*Surgical technique*

1. Preoperative management

After induction of general anesthesia and antibiotic prophylaxis with cefamezin 2 gr. (19), nasal cavities were packed with cottonoids containing epinephrine. Before surgery, a lumbar drain was placed and an intrathecal fluorescein infusion through the drain was performed, according to intra-hospital protocol (1 ml of CSF was withdrawn from the lumbar drain and mixed with 30 mg of fluorescein, and then slowly injected back into the lumbar drain). 

2. Patient positioning and tissue preparation for final reconstruction

**Fig 1:**
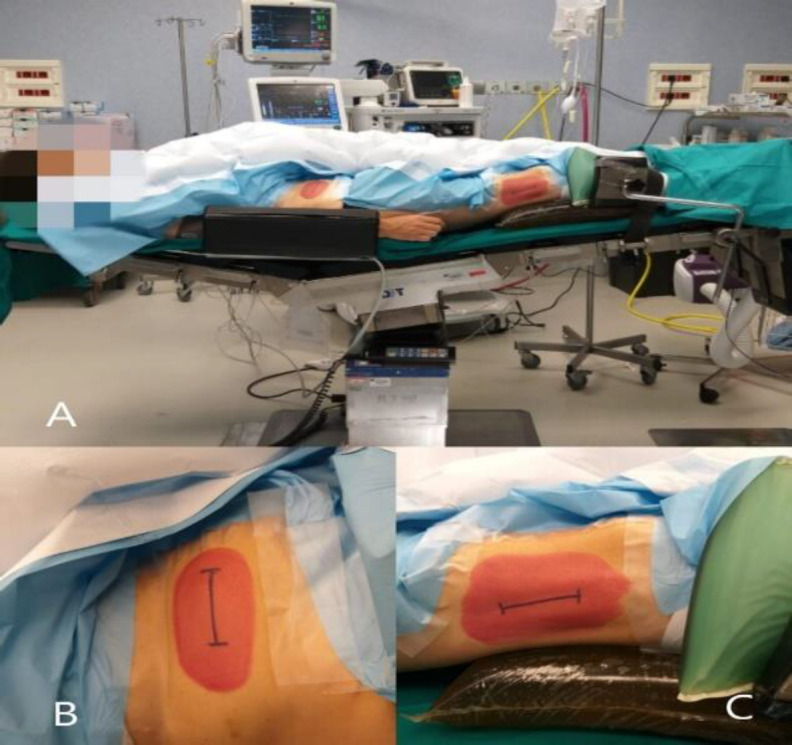
Patient positioning. A, the patient is placed supine with the head in neutral position and elevated to 10-30°; B, incision on abdomen in the right iliac fossa for autologous fat; C, incision on the right leg for ileo-tibial tract

The patient was placed supine with the head in neutral position and elevated to 10-30°. We used a 4K ultra-high definition endoscope (VISERA 4K UHD System; Sony Olympus Medical Systems, Tokyo, Japan) (20,21). The surgical approach was performed using arigid endoscopes of 0° (4 mm ULTRA Sinuscope; Olympus Medical Systems). Autologous fat tissue was taken from a small incision on abdomen in the right iliac fossa, while tissue for skull base reconstruction was obtained from ileo-tibial tract through an incision on the right leg (Figure 1). 

3. Approach step-by-step

*a. Antrostomy and sphenoidotomy description*

**Fig 2:**
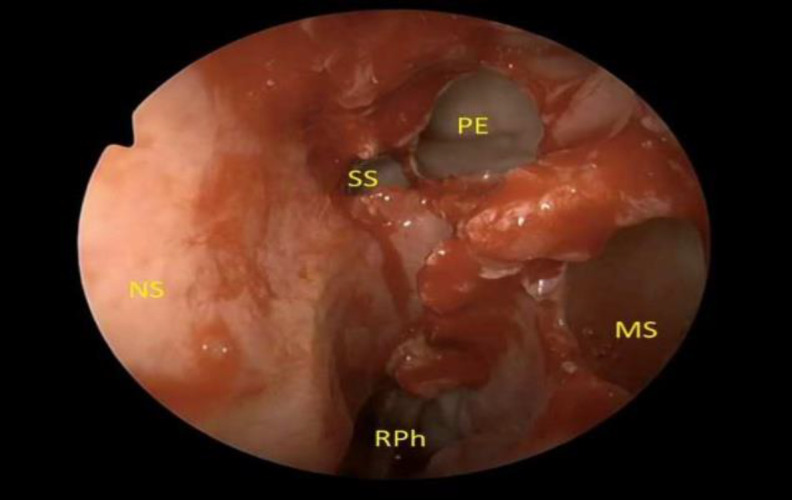
The picture shows the anatomy of the skull base during the preparation for a step-by-step left transpterygoid approach. The left uncinate process has been resected, and the left middle turbinectomy, maxillary sinus antrostomy, posterior ethmoidectomy and sphenoidotomy have been performed. NS, nasal septum, SS, sphenoid sinus; PE, posterior ethmoid; MS, maxillary sinus; RPh, right nasopharynx

The approach started with resection of left uncinate process, followed by identification of natural ostium of the maxillary sinus. The ipsilateral left middle turbinate was removed and the maxillary antrum opened, performing a middle turbinectomy and an antrostomy. A complete unilateral ethmoidectomy was then performed, resecting ethmoid bulla and extending to the posterior ethmoid. The posterior ethmoidal air cells were removed until sphenoid sinus was reached through a trans-ethmoidal route (Figure 2). 

The anterior sphenoidotomy was completed removing the sphenoid-ethmoidal septa, the anterior wall of the sphenoid sinus and the superior part of the vomer.

*b. Nasoseptal flap preparation and sphenopalatine foramenidentification*

**Fig 3:**
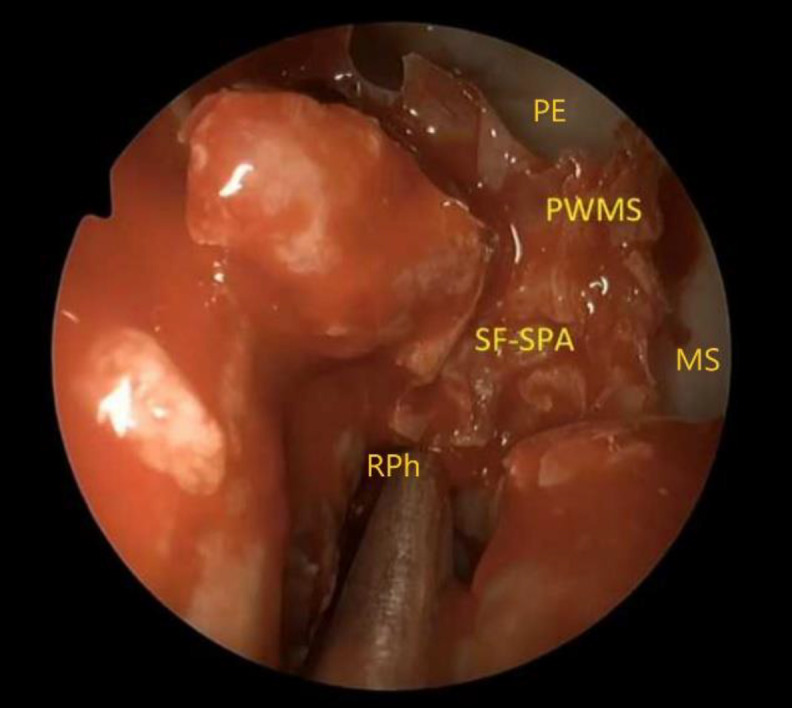
The ipsilateral septal flap with Hadad technique has been prepared, and the sphenopalatine foramen with the sphenopalatine artery have been localized. PE, posterior ethmoid; MS, maxillary sinus;SF, sphenopalatine foramen; SPA, sphenopalatine artery; PWMS, posterior wall of the maxillary sinus; RPh, right nasopharynx

The ipsilateral nasoseptal flap was prepared according to Hadad technique(1,6,22) described below. Two parallel incisions were performed on nasal mucosa of septum, the first over the maxillary crest and the second 1-2 cm below the superior limit of septum to preserve the olfactory epithelium. These two incisions were joined anteriorly by a vertical incision. Posteriorly, the superior incision was extended laterally over the rostrum of the sphenoid sinus crossing it at the level of the ostium, whereas the inferior incision was extended laterally crossing the posterior choana below the floor of the sphenoid sinus. The flap was then elevated using a Cottle dissector from its anterior part, dissecting up to the anterior face of the sphenoid sinus. Special care was done in the posterolateral area of the anterior face of the sphenoid sinus in order to preserve the neurovascular pedicle. The flap was stored into the maxillary sinus. The sphenopalatine foramen was localized behind the orbital process of the palatine bone, which constitutes its anterior limit (Figure 3).

*c. Sphenopalatine artery preservation*

In order to preserve the sphenopalatine artery, the orbital process and the medial part of maxillary sinus posterior wall removal started from the sphenopalatine foramen, achieving the exposition of pterygopalatine fossa contents. Bone removal proceeded laterally from the superior portion of the foramen toward the inferior orbital fissure and the foramen rotundum. The medial aspect of the pterygopalatine fossa and the greater palatine canal exposure (which contains the descending palatine artery and greater palatine nerve) was achieved removing the superior portion of the palatine bone perpendicular plate. The pharyngeal branch of the internal maxillary artery was identified in the palatosphenoidal canal (inferiorly and medially on the posterior wall of the pterygopalatine fossa) through the removal of the sphenoid process of the palatine bone.

*d. Access to* *pterygopalatine fossa and lateral recess of sphenoid bone*

**Fig 4:**
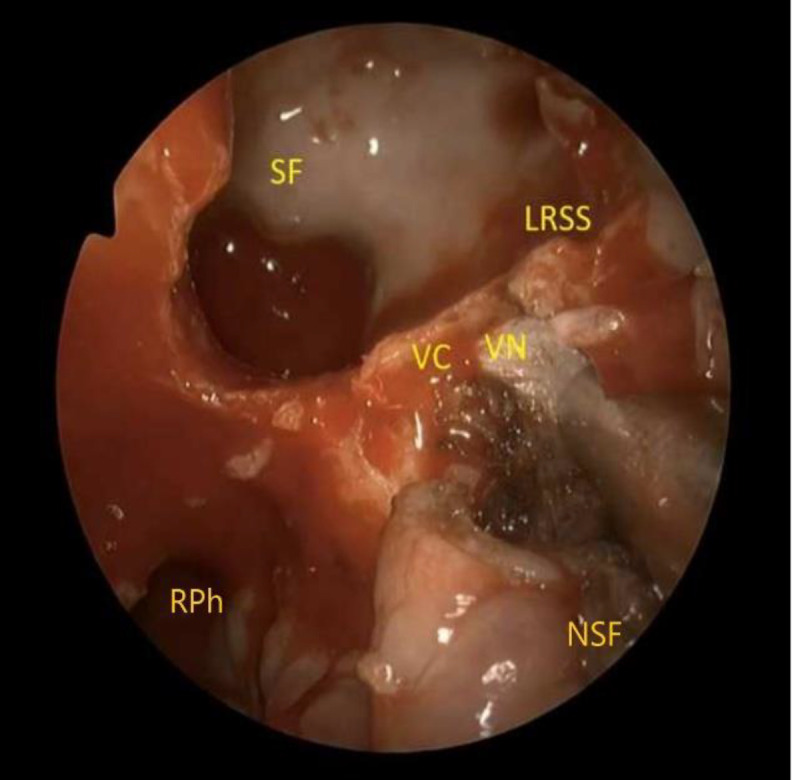
Thevidiancanal and the nerve have been isolated by removing the bone inferiorly and medially to the nerve, identifying the junction between the petrous and paraclival portion of the ICA. SF, sellar floor; VC, vidian canal; VN, vidian nerve; LRSS, lateral recess of the sphenoid sinus; NSF, nasoseptal flap; RPh, right nasopharynx

In order to obtain the lateralization of the pterygopalatine fossa contents, the pharyngeal branch was coagulated and cut. Subsequently, the palatovaginal artery was identified and dissected, while bone removal of vidian nerve canal proceeded starting inferiorly and medially to the nerve identifying the junction between the petrous and paraclival portion of the ICA (Figure 4). After drilling of vidian nerve canal, the vidian nerve was cut for further mobilization of the pterygopalatine fossa contents. The maxillary (V2) and the mandibular (V3) nerve were identified. 

*e. CSF leak identification and closure*

**Fig 5:**
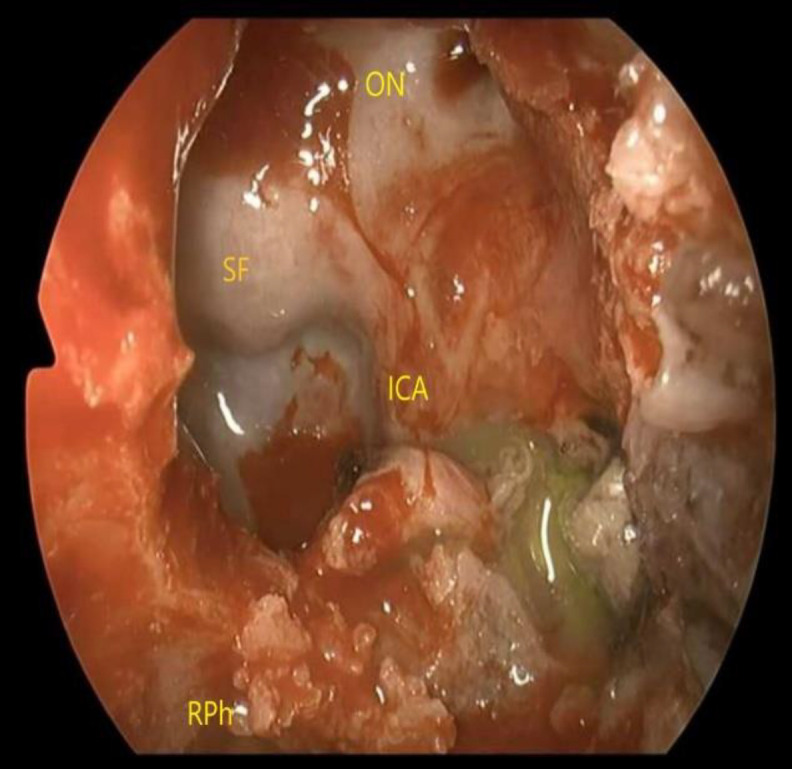
The CSF fistula and themeningocelehave been identified within the lateral recess of the sphenoid sinus through previous fluorescein injection.ICA, internal carotid artery; ON, optic nerve, SF, sellar floor; RPh, right nasopharynx

A CSF fistula and a small meningo-encephalocele were identified within left lateral recess of sphenoid sinus through previous fluorescein injection (Figure 5). 

**Fig 6:**
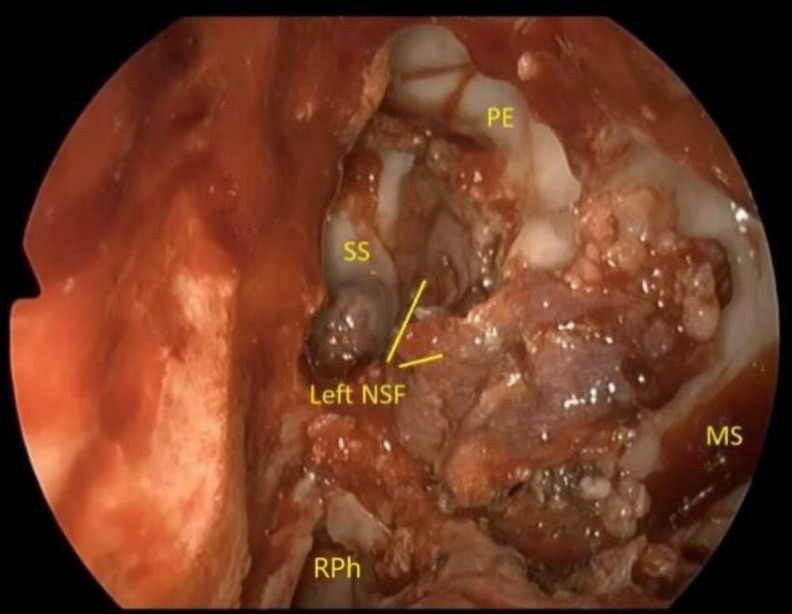
The picture shows the aspect of the reconstruction with a multilayer fashion at the end of the surgery. The reparation includes the apposition of autologous fat, fascia lata, and the transposition of the ipsilateral nasoseptal flap into the lateral recess of sphenoid sinus to cover the defect. SS, sphenoid sinus; PE, posterior ethmoid; Left NSF, left nasoseptal flap; MS, maxillary sinus; RPh, right nasopharynx

The meningo-encephalocele was coagulated and resected; the bony edges of the defect were prepared, coagulated and repaired in a multilayer fashion with autologous fat and fascia lata. Therefore, ipsilateral nasoseptal flap was transposed into the lateral recess of sphenoid sinus in order to cover the defect (Figure 6). 

The inferior incision along the choana should cross the medial pterygoid plate below the sphenopalatine foramen, in order to obtain enough tissue to cover the defect. The ipsilateral nasoseptal flap was secured using fibrin glue and pieces of Merocel placed on the edges of the flap. Finally, a non-absorbable anterior nasal packing with has been performed in order to facilitate mucosal healing ad avoid the displacement of skull base reconstruction materials (24). 

*f. Postoperative management*

The patient was admitted to the Neurosurgical ward after surgery. Lumbar drain was removed on postoperative day 3, while the anterior nasal packing was removed on postoperative day 4. During hospitalization, neurological examination and clinical evaluation to check postoperative CSF leak were performed daily. Patient was discharged on postoperative day 7 after an outpatient rhinoscopy. Further ORL outpatient rhinoscopies were performed at 30 and 90 days after surgery with evidence of good healing of nasal mucosa e no signs or symptoms of CSF leakage. 

## Discussion

The traditional endoscopic transsphenoidal approach is not adequate to expose diseases in the lateral recess of the sphenoid sinus or in the region of the sphenoid wing(8,12,16,23,24). Transpterygoid approach includes the partial or complete removal of the pterygoid process, allowing an extensive access to the skull base (infratemporal, middle and posterior cranial fossae). In fact, palatine bone is related with infratemporal fossa laterally, pterygopalatine fossa anteriorly, fossa of Rosenmuller and Eustachian tube posteriorly, and middle cranial fossa superiorly (3,5,8,10,24). 

Kasemsiri et al. (24) classified five types of endoscopic endonasal TA, based on the quantitative palatine bone removal. 

Type A consist in a partial removal of pterygoid process and it is indicated for pterygopalatine fossa diseases; Type B is characterize by removal of the anterior portion of the base of the pterygoid process, allowing the access to the lateral recess of the sphenoid sinus; Type C includes the removal of the entire base of the pterygoid process with dissection of the vidian canal for the access to the petrous apex and the Meckel’s cave; Type D is associated to the removal of the pterygoid plates and dissection of the petrous ICA. It is used to treat infratemporal fossa lesions with control of the petrous ICA. Type D includes also Eustachian tube removal, in order to manage extensive lesions extended to the middle and posterior cranial fossa (24). 

Thus, TA allows the possibility to minimize the amount of bone resection preserving the sphenopalatine artery, based on the extension of the pathology treated (23). Removing the orbital process of palatine bone and the postero-medial wall of maxillary sinus it is possible to achieve a complete visualization of the pterygopalatine fossa, which contains the internal maxillary artery (and sphenopalatine, descending palatine and postero-superior alveolar branches), the pterygopalatine ganglion, and the infraorbital nerve (8). Pterygopalatine fossa may be divided in an anterior vascular compartment, and a posterior neuronal compartment (10). In order to access to the lateral recess of sphenoid sinus, the contents of pterygopalatine fossa must be retracted laterally to achieve exposure of the lateral wall of the sphenoid sinus, thus allowing the removal of the sphenoid process of palatine bone.

In our experience, TA from type A to C leads to adequate access to the pterygopalatine fossa, lateral sphenoid recess, sphenoid wing and infratemporal region, allowing a successful skull base defect repair. The vidian nerve needs to be used as landmark for the ICA, and it can be sacrificed if necessary (3,8,10,22,24). Ulu et al. (16) promoted e minimal TA (Type B basing on Kesemsiri classification (24)) to treat sphenoid sinus lateral recess CSF leaks, observing an high successful rate, particularly if associated to nasoseptal flap. As in our experience, the possibility to use an ipsilateral nasoseptal flap if sphenopalatine artery is preserved during dissection was described. Schmidt et al.(8) suggested to preserve the ipsilateral sphenopalatine artery while performing TA, recurring to contralateral HBF only in cases of damage of the vascular pedicle. Pinheiro-Neto et al. (9) observed that a careful dissection of the sphenopalatine foramen removing the overlying bone is mandatory to avoid damage of the vascular pedicle. It is important to be familiar with the branches of the SPA and its anatomical variations. Authors observed that the bleeding should not be misinterpreted as injury to the flap pedicle, because of it could be related to injury of the palatosphenoidal branch of the internal maxillary artery (in case of bleeding in the roof of the nasopharynx), or damage of sphenopalatine artery supplying the inferior turbinate. 

They reported that vidian nerve preservation might limit the protection of the vascular pedicle when drilling the base of the pterygoid bone, suggesting vidian nerve scarification in order to achieve a simpler sphenopalatine artery preservation.

Kaligkiotis et al. (17) reported the use of ipsilateral flap during TA for the treatment of petrous apex lesions, suggesting to perform the inferior incision of the pedicle just above the tail of the middle turbinate, follow the choana and extend through the vomer to the maxillary crest, reaching the sphenopalatine foramen with both superior and inferior incisions. After the removal of orbital process and maxillary sinus’ posterior wall and pterygopalatine fossa opening, authors suggested to mobilize laterally its contents together with the periosteumin order to free the pedicle of the flap. Vidian neurovascular bundle and descending palatine and palatovaginal arteries transection could release the pedicle of the flap, increasing its length and mobility (17).

The use of ipsilateral nasoseptal flap during TA is very useful when multiple skull base defects, especially bilateral CSF leaks, are detected. In our opinion, in presence of bilateral TAs or combination of TA and other endoscopic approaches, an attempt to preserve both sphenopalatine arteries and prepare a nasoseptal flap on both side for skull base reconstruction should be done. Furthermore, if ipsilateral nasoseptal flap is achieved during TA, the controlateral HBF can be preserved in cases of re-operation (e.g. tumor or CSF fistula recurrence).

## Conclusion

A Transpterygoid approach with ipsilateral HBF for skull base reconstruction while preserving ipsilateral sphenopalatine artery is a possible surgical option and it can be considered for skull base reconstruction. The present article and the previous literature showed that deep anatomical knowledge, surgical expertise and careful dissection of neurovascular structures are fundamental to achieve wide exposure of pterygoid base without compromising the blood supply of ipsilateral nasoseptal flap. Furthermore, this technique can be used for skull base reconstruction in case of bilateral TAs or TA combined with other endoscopic approaches (i.e. CSF fistula of lateral recess of sphenoid sinus and extensive tumors) or for those cases with only one vascular pedicle available. 
